# P-405. Vancomycin Use in Critically-Ill Children with Heart Disease and Impact on Acute Kidney Injury

**DOI:** 10.1093/ofid/ofaf695.622

**Published:** 2026-01-11

**Authors:** Grant Stimes, Kajri Shah, Debra Palazzi, Claire Bocchini

**Affiliations:** Texas Children's Hospital, Houston, TX; Texas Children's Hospital, Houston, TX; Baylor College of Medicine, Houston, TX; Baylor College of Medicine, Houston, TX

## Abstract

**Background:**

Vancomycin (VAN) is a commonly used empirical agent for patients with concern for *Staphylococcus aureus* infection. VAN is a known nephrotoxic agent that can cause acute kidney injury (AKI) in at-risk patients such as children with heart disease who commonly are on concomitant nephrotoxic medications. The frequency of AKI in cardiac patients receiving VAN was the focus of this study.

**Methods:**

Patients receiving a course of VAN between 1/1/24 and 03/31/25 who were admitted to the Texas Children's Hospital cardiology floors were included. Each VAN course was defined as a separate encounter. Variables collected included: presence of cardiopulmonary bypass, duration of bypass, presence of ECMO, presence of renal replacement therapy (RRT) including modality and flow, presence of concomitant nephrotoxic medications, duration of VAN therapy, and detected pathogens in culture. Serum creatinine and urine output were recorded to identify and classify AKI using KDIGO criteria.

**Results:**

Of 146 VAN courses that met inclusion criteria, 130 occurred in patients without RRT and 21 had AKI (16%). In the 16 courses with concomitant RRT, 11 (69%) had AKI, most of which were in the patients receiving CRRT. Two of the 11 (18%) patients with AKI met severe AKI criteria. The majority (107/146) of VAN courses had at least 1 concomitant nephrotoxic medication administered. The overall median duration of VAN therapy was 40.02 hours (IQR 20.93-44.92 hours). Most (60%) patients received the standard 15 mg/kg dose. Ten patients received VAN after bypass with a similar duration of therapy (median 39.28 [IQR 34.77-43.67] hours); 4 of the 10 developed AKI (40%). Few (18; 12%) patients received VAN for definitive therapy; 7 (39%) grew a pathogen that required VAN for definitive therapy. Lastly, the presence of more than 1 concomitant nephrotoxin did not increase the RR of developing AKI 0.93 (CI 0.43-2.04).

**Conclusion:**

AKI occurred most frequently in critically ill children with heart disease receiving VAN with concomitant RRT. AKI was less frequent in those without RRT, even in the presence of concomitant nephrotoxic medications. VAN may remain a reasonable empirical agent for select children with heart disease and concern for *Staphylococcus aureus* infection.

**Disclosures:**

Debra Palazzi, MD, MEd, AHRQ: Grant/Research Support|AMA: Board Member|AMA: JAMA Pediatrics Associate Editor|Elsevier: UpToDate|PEW Charitable Trust: Grant/Research Support|University of Chicago/Lurie Children's: Honoraria

